# The lived experience of recovery in borderline personality disorder: a qualitative study

**DOI:** 10.1186/s40479-019-0107-2

**Published:** 2019-05-22

**Authors:** Fiona Y. Y. Ng, Michelle L. Townsend, Caitlin E. Miller, Mahlie Jewell, Brin F. S. Grenyer

**Affiliations:** 1 School of Health Sciences, Institute of Mental Health, University of Nottingham, Nottingham, UK; 2 School of Psychology, University of Wollongong, Wollongong, Australia; 3 Illawarra Health and Medical Research Institute, University of Wollongong, Wollongong, Australia; 4 Project Air Strategy Consumer and Carer Advisory Committee, Wollongong, Australia

**Keywords:** Borderline personality disorder, Recovery, Lived experience, Qualitative

## Abstract

**Background:**

The concept of recovery in borderline personality disorder (BPD) is not well defined. Whilst clinical approaches emphasise symptom reduction and functioning, consumers advocate for a holistic approach. The consumer perspective on recovery and comparisons of individuals at varying stages have been minimally explored.

**Method:**

Fourteen narratives of a community sample of adult women with a self-reported diagnosis of BPD, were analysed using qualitative interpretative phenomenological analysis to understand recovery experiences. Individuals were at opposite ends of the recovery continuum (seven recovered and seven not recovered).

**Results:**

Recovery in BPD occurred across three stages and involved four processes. Stages included; 1) being stuck, 2) diagnosis, and 3) improving experience. Processes included; 1) hope, 2) active engagement in the recovery journey, 3) engagement with treatment services, and 4) engaging in meaningful activities and relationships. Differences between individuals in the recovered and not recovered group were prevalent in the improving experience stage.

**Conclusion:**

Recovery in BPD is a non-linear, ongoing process, facilitated by the interaction between stages and processes. Whilst clinical aspects are targets of specialist interventions, greater emphasis on fostering individual motivation, hope, engagement in relationships, activities, and treatment, may be required within clinical practice for a holistic recovery approach.

**Electronic supplementary material:**

The online version of this article (10.1186/s40479-019-0107-2) contains supplementary material, which is available to authorized users.

## Background

Recovery in borderline personality disorder (BPD) has predominantly been viewed in the context of symptom improvement and no longer meeting diagnostic criteria. Longitudinal studies have demonstrated that symptom remission is a common occurrence, with remission rates ranging between 33 and 99% [1]. Personal recovery however, adopts a holistic stance and views recovery as a process rather than a fixed outcome [2, 3]. Conceptual frameworks of personal recovery have synthesised the stages across the transtheoretical model of change, and processes into the CHIME framework (connectedness, hope, identity, meaning and empowerment) [4]. The application of personal recovery to individuals with BPD requires further exploration [5].

Qualitative studies examining the experience of individuals with personality disorder describe recovery as involving the reconciliation of self and other representations, fostered through interpersonal relationships and integration within the community [6, 7]. These views were similarly identified by Castillo and colleagues [8] who described recovery as a hierarchical process, starting from the development of healthy attachment patterns, progressing to a state of transitional recovery. This process encompassed stages including, the sense of belonging, and development of hope, goals, identity and roles [8]. These stages were similar to the personal goals by Katsakou and colleagues [2], which included aspects associated with regulating emotions and other symptoms. These findings were further confirmed in a study of treatment goals of individuals seeking treatment for BPD, where goals were identified to extend beyond the reduction of symptoms and included improving relationships, developing a sense a self and improving one’s sense of wellbeing [9]. Whilst these findings indicate the treatment targets of manualised interventions may be narrow, there are innate difficulties in understanding recovery in personality disorders [7], given the similarities between clinical phenomenology and domains of personal recovery. The current changes to the conceptualisation of personality disorder from a categorical to dimensional approach, focusing upon individual traits, severity, and functioning, provides an opportunity to more fully integrate individual perspectives into treatment [10].

The perspectives of individuals accessing specialist treatment have been well represented within the literature. While important, a broader approach to include individuals who do not access specialist services, such as who have difficulty accessing services or no longer require services may provide a more representative view. This coincides with calls to further understanding the experiences of people who are at the opposite ends of recovery [11]. Therefore, this study aims to understand the experience and conceptualisation of recovery in individuals with BPD who are at varying stages of the recovery process. Comparisons between individuals in the recovered and not recovered groups were made to illustrate differences.

## Method

### Participants and inclusion

Individuals were initially recruited to take part in an online survey, via mental health organisations and social media, adapting methods used by previous studies of experience in personality disorder [12]. The study’s inclusion criteria was based on the recognition in the wider literature that recovery may occur across stages and is fluctuating in nature (Andresen et al., 2003). A longitudinal study of individuals with schizophrenia identified that half the sample did not progress past the first stage (‘overwhelmed by the disability’), and no individuals attained the final stage of recovery (‘living beyond the disability’) within the two-year follow-up period [11]. Findings from a study examining recovery in BPD similarly identified the final stage (‘recovered’) to be more uncertain [2]. Therefore, the perspectives of individuals at the extreme ends may be important to understand in order to capture what the recovery spectrum in BPD may entail.

Following completion of an online survey, researchers grouped individuals into one of four groups identified by recovery and diagnostic status. Recovery status was obtained through asking individuals to define recovery in BPD and identification with their personal definition. Diagnostic status was determined through the McLean Screening Instrument for Borderline Personality Disorder (MSI-BPD) [13]. The MSI-BPD is a 10-item self-report screening measure, where a score of 7 or greater indicates the high likelihood of meeting DSM-5 criteria for BPD. The MSI-BPD has good psychometric properties with high sensitivity (0.81), specificity (0.85) and reliability (alpha = 0.74) [13]. The narratives of individuals who self-identified with being recovered and no longer met criteria for BPD (recovered group), and individuals who did not self-identify with being recovered and met criteria for BPD (not recovered group) were included in the study. Individuals were further matched on age, gender, and treatment history. Narratives were included into the study until thematic saturation was reached. This resulted in the inclusion of 14 individual narratives (*n* = 7 recovered group and n = 7 not recovered group). The study was approved by the University of Wollongong Social Sciences Human Research Ethics Committee (HE16/215) and all individuals provided informed consent.

### Data analysis

Semi-structured interviews following a topic guide were conducted. The guide provided general prompts for the interviewer and was refined following consultation with a consumer advisory committee (Additional file 1). The interviewer asked individuals to describe their first experiences with BPD, current life, views of recovery, and experience of treatment and supports. Interviews were audio recorded, transcribed verbatim and entered into NVivo 11 for data analysis.

Interpretive phenomenological analysis (IPA) was used as the overarching methodology to understand individuals’ experience and the ascribed meaning associated with the recovery journey in BPD [14]. Smaller sample sizes are recommended to gain in-depth understanding [14]. An inductive approach outlined by Smith and colleagues [14] was used to understand the emergent themes and the relationship between themes. Firstly, researchers immersed themselves in the narrative by reading transcripts, whilst free coding to gain an overarching understanding of the data. Secondly, free codes were coded into emergent themes summarising excerpts of individual’s narratives. Emergent themes were then clustered into superordinate themes to describe individuals’ experiences. This process was supported by discussions by the research team, where discrepancies between the team were resolved via consensus. Two transcripts, which represented over 10% of the data were coded by two independent raters (FN and CM) (inter-rater reliability = 91%). The remaining data was independently coded by one researcher (FN). The names of individuals have been de-identified to their participant number for confidentiality purposes. Individuals in the recovered group are denoted with ‘R’ and those who are not recovered are denoted with ‘NR’. Once the coding was determined by the researchers, the findings were discussed with a member of the consumer advisory committee, whose feedback was integrated to strengthen the paper (MJ).

## Results

A total of 171 individuals provided contact details for follow-up from the online survey, where 108 individuals were contacted. Thirty-nine individuals completed the telephone interview. Using the study’s inclusion criteria, 14 individual narratives (7 recovered and 7 not recovered) were included in the study. All individuals in this study were female with an average age of 33.36 years (SD = 10.26). The majority of individuals were from Australasia, with one individual from the Middle East. There were no significant differences on socio-demographic characteristics between the two groups. Comparison of socio-demographic characteristics of individuals are provided in Table 1.

**Table 1:** Comparison of Socio-Demographic Participant Characteristics

Variable	Statistic	Total (*N* = 14)	Recovered Group (*n* = 7)	Not Recovered Group (*n* = 7)	t (p) or χ^2^
Age	M (SD)	33.36 (10.26)	33.43 (11.43)	33.29 (9.88)	0.03 (0.98)
	Range	R = 18–52	R = 18–52	R = 22–46	
Education (years)	M (SD)	14.29 (1.94)	14.29 (2.29)	14.29 (1.70)	0.00 (1.00)
	Range	R = 11–16	R = 11–16	R = 11–16	
Employment status					
Engaged in paid work	% (n)	42.9 (6)	42.9 (3)	42.9 (3)	1.14 (0.57)
Relationship Status					
Single	% (n)	64.3 (9)	57.1 (4)	71.4 (5)	0.31 (0.58)
In relationship	% (n)	35.7 (5)	42.9 (3)	28.6 (2)	
Treatment Length (years)	M (SD)	12.68 (6.72)	9.93 (9.47)	15.43 (5.00)	−1.63 (0.13)
	Range	R = 2–24	R = 6–15	R = 9–21	
Age of Onset	M (SD)	9.71 (3.58)	10.57 (3.55)	8.86 (3.67)	−0.36 (0.73)
	Range	R = 4–15	R = 6–15	R = 4–14	
Age of Diagnosis	M (SD)	25.57 (10.14)	24.57 (9.47)	26.57 (11.44)	0.89 (0.39)
	Range	R = 15–45	R = 16–44	R = 15–45	
Gap between Onset and Diagnosis	M (SD)	15.86 (10.88)	14.00 (10.79)	17.71 (11.50)	−0.62 (0.55)
	Range	R = 6–38	R = 7–38	R = 6–34	

Note. No significant differences between recovered and not recovered groups

### Stages of recovery in borderline personality disorder

**Fig. 1:**
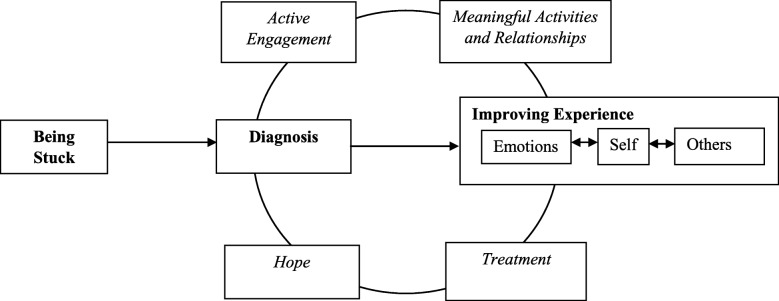
Stages and processes of recovery in borderline personality disorder

Recovery in BPD occurred across three core stages, including; 1) being stuck, 2) diagnosis, and 3) improving experience. Differences between individuals in the recovered and not recovered groups were observed in the final stage of recovery continuum. The movement between stages fluctuated, therefore narratives were discussed from a current or retrospective stance. A graphical representation of the stages and processes of recovery in BPD is depicted in Fig. 1.

### Being stuck

This stage was characteristic of all individuals when first experiencing symptoms of BPD. Individuals did not have a clear conceptualisation of their experiences and described ‘being stuck’ as a state of *‘floundering, getting bounced in and out of hospital… I was lacking in therapy and not really engaging in services’* (JTR191-R). An individual’s emotional intensity was identified to impact upon daily living and was noted to extend beyond the realms of normal experience, where *‘emotions are so raw and powerful, they drove everything. I had no insight whatsoever into what I was doing. I didn’t know who I was, what I was doing or why. I reacted to everything in an unhealthy way’* (JTR280-R). Reports of maladaptive coping strategies such as self-harm or repeated suicide attempts were prevalent at this stage.

Negative experiences from childhood and adolescence, such as bullying or abuse, was reported to affect an individual’s perception about self and others. For example *‘BPD can be rooted in childhood trauma… I was taught it was always my fault as a child. Being in a relationship now with the same thing happen, my brain will assume, it is my fault’* (JTR051 – NR). The enduring nature was also noted in interpersonal difficulties, such that *‘even at six years old, I had that instable personality… Not having any kind of self-worth and switching from one friend to another depending on what my needs were and how that person was feeling…’* (JTR239 – R).

Unsuccessful attempts at seeking help for mental health concerns was also characteristic during this stage. Misdiagnosis of other mental health concerns, such as depression, anxiety and bipolar disorder, were a common experience. Individuals reported these diagnoses did not encapsulate the severity of their experience, as *‘it felt much worse but they told me my problems are mild and not an issue’* (JTR051 – NR). The knowledge of health professionals and the ability to access effective treatments were viewed to be crucial for an individual to move on from the ‘being stuck’ phase.

### Diagnosis

Receiving a diagnosis of BPD was identified to be a turning point in assisting individuals to conceptualise their experiences and emotional intensity. Diagnosis provided individuals a narrative *‘to describe what was going on, that I wasn’t alone and other people had experienced this as well’* (JTR011 – R), giving individuals a sense of validation and relief, which assisted with progression in the recovery journey. The impact of delayed or mis-diagnosis was highlighted in the length of time taken to receive a diagnosis of BPD, as diagnosis assisted some individuals to gain access to evidence based treatment for BPD. Non-acceptance or disinterest in the diagnosis was reported by a minority of individuals, *‘I didn’t accept the borderline diagnosis. I wasn’t interested and no one was interested in talking to me about it… but I understood what bipolar was and thought that did seem to fit’* (JTR239 – R). Some participants highlighted the immediate need for information about BPD to contextualise the diagnosis, as *‘the worst thing is when people are not given any information when they are diagnosed with BPD.’* (JTR280 – R). Whilst knowledge was predominately acquired from engagement with health services, some individuals identified their own efforts to gain knowledge, *‘I did a lot of reading once I got the diagnosis. It really made sense’* (JTR011 – R). However, the prevalence of stigma and discrimination associated with the diagnosis of BPD promoted negative experiences, where *‘I’ve had some really traumatic experiences as a result of having the diagnosis… I no longer seek help if I’m in crisis, because I know that I’ll get treated badly and be more stressed than if I didn’t do anything...I feel like I don’t trust the system’* (JTR051 – NR).

### Improving experience

Developing greater awareness of emotions and of self and others was described as a core stage and influencer of recovery. Three domains were associated with this stage including 1) Developing Greater Awareness of Emotions and Thoughts, 2) Strengthening Sense of Self, and 3) Understanding the Perspectives of Others. These domains were not mutually exclusive, yet the progression made in this stage differed between individuals.

Individual’s conceptualisation of recovery indicated that there was skepticism surrounding the amelioration of symptoms. Recovery was considered an ongoing journey with elements of survival, resilience and self-management. For example, *‘it can be managed… I don’t think the symptoms will ever 100% disappear forever. They’ll always be there to some degree in the background. I hope I get to a point where it doesn’t impact on your life in a negative way’* (JTR051 – NR). This was echoed by individuals who identified with being recovered as, *‘I got to a point where I realised that all that suffering made me much stronger. I have more insight because I had to do the work to recover’* (JTR280 – R).

### Developing greater awareness of emotions and thoughts

The identification of emotions and thoughts was considered a starting point in fostering understanding of oneself and the use of coping strategies, such that *‘I was beginning to develop more awareness of my emotions, but not so much control. Just the ability to not be blindsided by them’* (JTR459 – NR). However, the identification of emotions did not preclude individuals to distress, where *‘I don’t necessarily act on my thoughts anymore. My first reaction to something will be ‘I should self-harm’, but even though I’m not actually physically doing it, having my thoughts consumed by it is distressing’* (JTR083 – NR).

### Strengthening sense of self

All individuals acknowledged that developing one’s sense of self was a central component of the recovery journey. Individuals who identified with being recovered provided greater details of the nuances of developing a stronger sense of self. This was conceptualised as a process of reframing how one understands or perceives oneself. This process was noted to commence in conjunction with developing skills to recognise and tolerate emotions.

Individual narratives discussed the lack of identity stemming from first experiences of BPD and their sense of self being constructed upon symptom experience and identification with the BPD diagnosis. For example; *‘Sometimes I feel like my whole identity has been based around my trauma… and when you suddenly start being able to react differently to things, I kind of felt like a lot of my identity was disappearing, because I no longer feel as intense’* (JTR051 – NR). Stigma arising from interactions with others had the potential to reinforce negative self-perceptions, such that *‘I was very reluctant to actually disclose to people [my diagnosis] up until only really a few years ago, because I disclosed previously without thinking about it and then experienced unpleasant responses.’* (JTR011 – R).

Being aware of individual patterns and triggers provided opportunities to *‘always challenge myself to become better. Instead of avoiding things like I used to, I think about how I can do it until I’m not stressed out by it anymore’* (JTR233 – R). This allowed for skill practice but also a subsequent sense of agency. Difficulties moving away from the illness identity was articulated by a minority of individuals in the recovered group. Despite progress made in identifying emotions and skill usage, individuals noted that *‘my therapist had been telling me that I was recovered and I didn’t meet criteria, but I didn’t believe her. I think it was because I lacked an identity. I still don’t understand what identity is… I held onto that diagnosis for such a long time, that was who I was’* (JTR239 – R). The fear associated with developing a greater sense of self exacerbated this as *‘what if I use the skills and do what I need to do to achieve recovery and I still hate myself?’* (JTR280 – R).

### Understanding the perspectives of others

This theme was discussed by a minority of individuals in the recovered group. Individuals described this as a process of reflecting beyond one’s own subjective experience to include the capacity of others and the relational context. The impact of being able to understand the perspectives of others in reconciling relationships was highlighted in an individual’s response, where *‘I got to experience the pain that I inflicted on my mother, by projecting all my self-loathing onto her. My mum had her own weaknesses… but I was too caught up in my own narcissistic injuries before to conceptualise how much pain I’d caused her.’* (JTR191 – R). This was similarly discussed by another individual, where the perspectives of others allowed for the calibration of her own perceptions of self. For example *‘My husband always saw my potential and knew what I’m capable of, but I didn’t see that at the time. I just thought he was ridiculous and was making fun of me, but I now know what he means’* (JTR072 – R).

### Processes of recovery in borderline personality disorder

Four recovery processes in BPD were identified from individual’s narratives; 1) active engagement in the recovery process, 2) hope, 3) treatment and, 4) meaningful activities and relationships. These processes could be overlapping and facilitate or hinder the recovery journey. Some differences between individuals in the recovered and not recovered groups were identified. These recovery processes contributed to the movement through the recovery stages and the growth within individuals.

### Active engagement in the recovery process

The desire and willingness to engage in the recovery process was crucial for progress in recovery to be made. Yet these observations were often made from a retrospective standpoint, when individuals had already accepted their diagnosis and take *‘responsibility to learn the skills and do it yourself, you’re going to get to a finite point, where it’s all going to be ok’* (JTR011 – R). Motivational differences between individuals in the recovered and not recovered groups were identified, such that individuals in the recovered group placed emphasis on intrinsic factors, whilst individuals in the not recovered group emphasised extrinsic factors. A minority of individuals identified that the mindset in which they approached treatment may impact on willingness to active engage in recovery such that a change-oriented mindset was necessary. *‘I was in treatment but I thought why I was sitting there listening to other people talk about their issues. I thought this isn’t my problem and I felt so angry, I didn’t see the point, so I dropped out.’* (JTR239 – R).

### Hope

Hope was an overarching concept, permeated when experiences positively contrasted to individual perceptions or their worldview. Recovery was considered unexpected and promoted a new outlook which was not previously considered by some individuals. States of hopelessness particularly observed during the early stages was prevalent in all individuals, such that *‘I didn’t have any kind of hope. I didn’t have anything to hold onto…’* (JTR239 – R). Hope could be generated through vocational and relational engagement and the subsequent sense of agency gained from the use of skills or reflection on progress. For some individuals in the not recovered group, the maintenance of hope was associated with the ability to get treatment, *‘I had a wonderful psychologist who I got along really well with. But at the moment it’s hard to keep my eye on the prize,* per se*’* (JTR459 - NR).

Hope played a role in the maintenance of motivation, as it contributed to gains in self-belief and the reduction of self-doubt. *‘That sense of just knowing the emotions will end, this isn’t a permanent thing... I used to feel like it was just never going to end’* (JTR239 – R). The shift in perspective had a compounding effect on individuals and their clinicians, as *‘…I suppose I wouldn’t expect it (recovery). I mean my clinicians were surprised by my recovery’* (JTR151 – R).

### Engagement with treatment services

Seeking treatment was identified by all individuals as a key component in the recovery process, where effective treatment aligned with individual goals provided a sense of hope and the development of skills. Whilst these provided individuals a sense that *‘this could be working. Maybe things will be ok’* (JTR061 – NR), services and treatments were described as mixed and fragmented. All individuals described at least one negative experience, where difficulties accessing treatment hindered progress on recovery. Individuals described greater difficulties when at the start of the recovery continuum.

Incongruent relationships through a lack of therapeutic alliance between clinician and individual also contributed to a lack of progress made in recovery, such that *‘I don’t think I progressed much with them (clinician) because we didn’t fit well’* (JTR051 – NR). This contrasted to the progress made with clinicians who promoted collaborative and trustworthy relationships, as these fostered stronger relationships, as *‘she would make an appointment with me and I wouldn’t turn up. She didn’t get angry… she just kept trying and waited until I was ready’* (JTR233 – R).

### Engaging in meaningful activities and relationships

Engaging in meaningful activities and relationships was described as providing a sense of belonging and connectedness, the opportunity to practice new skills, reflect upon one’s emotional reactions and sense of self. Although individual differences influenced what was considered meaningful, these commonly included employment, education, and relationships with friends, family, significant others and clinicians. Benefits such as the independence gained from being employed and the sense of *‘affirmation and sense of purpose’* (JTR011-R) was discussed.

For some individuals during the early experiences of BPD, their experience of symptoms precluded their participation in activities such that when *‘when I was a student and before I started working full-time, it was much harder and my symptoms were more pronounced. I had a lot more difficulty’* (JTR011-R). This also extended into the relational domain, where some individuals avoided relationships in fear of the negative effects on symptoms, such that *‘I haven’t had a relationship for the last seven months, it’s easier when you don’t have one… I’m really scared of actually going into a relationship again, because when that goes bad, I’m going to go bad.’* (JTR018 – NR).

All individuals acknowledged the role activities and relationships had for self-exploration and reflection. For example, meditation was described by one individual as *‘a laboratory that helps you sit with yourself and watch how the emotions just rise and fall away’* (JTR191 – R). Whilst others identified differences in self in differing contexts, for example *‘At work I would be fine, but I can be a complete mess outside of work. I can organise 10 other people but then my brain just switches. As soon as I don’t have something to focus on, I focus on myself, which is bad.’* (JTR018 – NR). Noticing differences in oneself provided opportunities to gain greater insight into oneself.

## Discussion

The present study aimed to gain a holistic understanding of recovery in individuals with lived experience of BPD at either end of the recovery continuum. Overall, recovery was characterised by an interaction between the stages and processes. The identification of recovery in BPD as an ongoing journey is reflective of current literature on personal recovery in mental health [2, 15].

The stages of recovery identified in the present study align with the broad recovery stages mapped by Leamy and colleagues [4]. However, stages identified were framed by individuals in a clinical manner. Domains associated with improving experience were reflective of core psychopathology in BPD [16]. This mimics the tasks identified in other qualitative studies examining recovery in personality disorder [2, 6, 7]. Therefore, the developed framework may be reflective of recovery within the context of treatment. Individuals in this study on average had 10 years of treatment, therefore the importance of treatment as part of recovery is not without standing. Yet, the literature proposes that there are multiple routes to recovery, including engagement in non-traditional mental health services [3], The possibility of individual recovery through the use of other supports, such as peer workers or recovery colleges, could be further investigated within the context of personality disorder. Despite this, only the perspectives of women were included as part of the study. The perspectives of men could be a focus for future research.

As individuals were required to have a diagnosis of BPD to take part in the study, the being stuck and diagnosis stages were universally described. Diagnosis played a role in shifting the trajectory of experience and provide opportunity to formulate meaning and promote hope. However, the gap between an individual’s perceived age of onset and age of diagnosis in this sample was approximately 15 years. This may be representative of a knowledge gap in health professionals and the need to upskill clinicians in working with people with personality disorder or stigma which may prevent timely diagnosis [7, 17, 18]. This compounds with the desire of individuals for information about BPD at diagnosis.

Differences between the recovered and not recovered groups were most pronounced in the improving experience stage. The narratives of individuals in the recovered group articulated experiences of understanding self and others, compared to individuals in the not recovered group who discussed working towards improving awareness of emotions and thoughts. Whilst growth is exemplified as a stage in other models of personal recovery, often involving self-management of symptoms [19], narratives in this study indicate that the process of growth began through gaining awareness of emotions.

Strengthening the sense of self was identified to be a domain central to growth. There are differences between what is currently conceptualised as identity in the personal recovery literature, which proposes that individuals reformulate their sense of self [20, 21], suggesting that individuals have some sense of self, prior to their first experiences of mental health concerns. In this study, individuals describe a lack of identity from first experiences of BPD. Adopting an illness identity has been associated with less favourable outcomes [22], whilst the movement away from illness identity is supported by the current personal recovery literature [4]. The emphasis on diagnosis in the current findings suggests that acceptance of the illness is required to a degree to progress in recovery in BPD. Over-identification however, can also lead to stagnation in recovery. Greater understanding of illness identity in BPD is required and is particularly salient given identity disturbances is core to the disorder. Identifying internal narratives may be a starting point in promoting motivation and willingness to engage in the recovery journey.

Engaging in relationships and meaningful activities is known to be a priority for individuals with BPD [1]. Interestingly, the proportion of individuals engaged in paid employment and in a relationship did not significantly differ, despite individuals being at either end of the recovery continuum. This indicates that recovery status may have an influence on the quality of the relationship or the amount of work engaged in. Existing longitudinal studies have identified that approximately 50% of individuals experience ‘good recovery’ following 10 years of follow-up, indicating that individuals have experienced concurrent remission from BPD and have full time vocational engagement [23, 24]. In the present study, less than half of the individuals in the recovered group were engaged in a relationship or in paid work, indicating that the current sample may have a more severe presentation and experience greater psychosocial difficulties compared participants in existing longitudinal studies. Differences between the treatment context in individuals in the current sample and longitudinal studies such as The McLean Study of Adult Development [24] are worth noting. Individuals in the McLean study were more functional and therefore likely to be employed than those in the current sample. This may be due to differences in capacity to pay for and access care, with the McLean sample being mainly health insured patients compared with our sample that were more reliant on stretched public services for care.

The broad recruitment strategy adopted by the study allowed for individuals to be recruited from more than one treatment service or service catchment, allowing for a wider range of views and experiences to be included in the study. However, consistent with previous research, the study adopted a retrospective approach. Difficulties in comparing individuals were encountered by researchers, as recovery is not a static process. For example, individuals in the not recovered group may have previously experienced periods in which they considered themselves as recovered and could draw on these experiences. The narratives of individuals may be subjected to some level of response bias given the significant gap between individual’s age of onset, diagnosis and current age. The use of prospective longitudinal research to map recovery to obtain real time accounts may be a direction for future research. The adoption of blind data collection and analysis process may also reduce the likelihood of researcher bias.

## Conclusion

This study identified stages and processes associated with recovery in BPD through the perspectives of individuals with lived experience. The findings extend existing knowledge by contrasting the experiences of individuals at either ends of the recovery continuum. The inclusion of individuals in the recovered group, provides a stronger indication of what the full recovery spectrum may constitute. The findings however, represents recovery in the context of treatment. Therefore, it is difficult to extend these findings to individuals who seek support for BPD outside of traditional treatment services. To incorporate a more holistic approach to recovery in clinical practice, it is recommended that a greater focus on individual motivation, treatment engagement, relationships and hope is needed.

## Additional file

Additional file 1: Interview Schedule. (DOCX 15 kb)
