# Supplementary material for: The lived experience of recovery in borderline personality disorder: a qualitative study
Source: Borderline Personal Disord Emot Dysregul. 2019 May 22;6:10. doi: 10.1186/s40479-019-0107-2 (PMC6532193; doi:10.1186/s40479-019-0107-2)
Supplement: Supplementary file 1 — Interview Schedule. (DOCX 15 kb) [file 40479_2019_107_MOESM1_ESM.docx]

**Online Supplement 1**

Interview Schedule

1. Can you describe your life at the present moment?
2. Do you think you are on a journey towards recovery? Why or why not?
3. The next few questions are about what your life has been like for you since you started noticing difficulties with BPD

*Prompts*

- 1. Thinking back to when all this started, what did you experience when you first started noticing that you were having difficulties with BPD?
  2. What was your life like then?
  3. Did you notice your difficulties or did someone else?
  4. Then what happened?
  5. How long did these periods in your life last for?
  6. How does your life differ now compared to when you first noticed your difficulties?

1. What would recovery look like to you?
   1. How would you know when you have reached that stage?
   2. At which stage/point do you consider yourself at the present moment? (and why)
2. Reflecting on the treatment and support you have had so far, what has been the most helpful and least helpful aspects so far?

*Prompts:*

- 1. What were the most important factors that helped you?
     1. In terms of: services, people, peers
  2. What you do think contributed to the helpfulness of services/people/peers etc?
  3. Were there aspects of your life (other than treatment) that you feel like is contributing to getting well?
  4. In what ways do you think you could have been more supported in getting well?
     1. In terms of: Services, people, peers
  5. If you had to summarise what has helped you the most so far, what are the top five factors?

1. Is there anything else that you think is important that we haven’t discussed that you would like to add?
